# Upregulation of ARNTL2 is associated with poor survival and immune infiltration in clear cell renal cell carcinoma

**DOI:** 10.1186/s12935-021-02046-z

**Published:** 2021-07-03

**Authors:** Song Wang, Xueyou Ma, Yufan Ying, Jiazhu Sun, Zitong Yang, Jiangfeng Li, Ke Jin, Xiao Wang, Bo Xie, Xiangyi Zheng, Ben Liu, Liping Xie

**Affiliations:** Department of Urology, School of Medicine, The First Affiliated Hospital, Zhejiang University, 79 Qingchun Road, Hangzhou, 310003 Zhejiang China

**Keywords:** ARNTL2, Clear cell renal cell carcinoma, Unfavorable survival, Immune infiltration, xCell

## Abstract

**Background:**

Aryl hydrocarbon receptor nuclear translocator like 2 (ARNTL2) is a member of the PAS superfamily. Previous studies explored the carcinogenic roles of transcription factor ARNTL2 in human malignancies. However, its roles in ccRCC have not been elucidated. This study sought to explore the roles of ARNTL2 in ccRCC and determine its correlations with tumor immunity.

**Methods:**

The expression of ARNTL2 was analyzed using the GEO, TCGA and GTEx database, and verified in ccRCC tissue samples and cell lines by qRT-PCR and western blot analysis. Kaplan–Meier survival curve analysis, Cox regression analysis (including univariate and multivariate analysis) was utilized to evaluate the prognostic values of ARNTL2. Potential biological mechanisms of ARNTL2 were explored using GSEA method. Colony formation and wound healing assays were conducted to explore the oncogenic role of ARNTL2 in ccRCC. ssGSEA and xCell algorithm were used to explore the correlation between ARNTL2 expression and tumor immune microenvironment (TIME).

**Results:**

ARNTL2 was significantly upregulated in ccRCC tissues and cell lines compared to normal kidney tissues and cell line. Enhanced expression of ARNTL2 was strongly linked to advanced clinical stage and unfavorable overall survival in ccRCC. ARNTL2 was determined as an independent prognostic marker through cox regression analysis. A prognostic nomogram was constructed to predict 1-, 3- and 5-year overall survival of ccRCC patients by integrating ARNTL2 expression with other clinicopathologic variables. GSEA analysis showed that focal adhesion, T cell receptor, cell cycle, and JAK-STAT signaling pathway were significantly enriched in high ARNTL2 samples. Silencing of ARNTL2 suppressed the colony formation ability and wound healing efficacy of ccRCC cell lines. xCell analysis showed that high expression level of ARNTL2 exhibited an immune infiltration status similar to CD8 + inflamed ccRCC subtype, which was characterized by high infiltration level of CD8 + T cell and high expression level of the immune escape biomarkers such as PD-L1, PD-L2, PD1 and CTLA4.

**Conclusion:**

ARNTL2 is an independent adverse predictor of ccRCC patient survival. High expression level of ARNTL2 is associated with immune infiltration, and may be a novel therapeutic target in ccRCC.

**Supplementary Information:**

The online version contains supplementary material available at 10.1186/s12935-021-02046-z.

## Background

Kidney cancer is among the top ten cause of cancer-related deaths worldwide. Kidney cancer results in more than 431,000 new cases and 179,000 deaths annually. Clear cell renal cell carcinoma (ccRCC) is the most common subtype of kidney cancer [1, 2]. Despite significant advances in modalities of diagnosis and treatment in the recent decades, ccRCC is a major threat to health and significantly affects the quality of life of patients worldwide, mainly metastatic patients that benefit few from currently available therapies [3]. Studies reported that interactions between cancer cells and immune system play a vital role in carcinogenesis of ccRCC [4, 5]. Meanwhile, current immunotherapies, such as monoclonal antibodies targeting PD-L1 and/or CTLA4, have become the main therapy for advanced ccRCC treatment [6]. Based on the CheckMate025 study [7], Nivolumab was identified as the first PD-1 monoclonal antibody approved for the treatment of advanced ccRCC, which significantly prolonged the overall survival of ccRCC patients. Subsequently, a series of clinical trials demonstrated that Nivolumab in combination with Ipililmumab was superior to targeted therapy in patients with advanced ccRCC [8, 9], ushering in a new era of first-line treatment for advanced kidney cancer. However, there are still many patients who benefit less from immunotherapy as they are not sensitive to these immunotherapy agents. Therefore, there is need to discover effective prognostic biomarkers and highly specific tumor immune related therapeutic targets to improve treatment of ccRCC patients.

Aryl hydrocarbon receptor nuclear translocator like 2 (ARNTL2) belongs to the PAS superfamily. It encodes a transcription factor, whose protein structure is highly homologous to hypoxia-inducible factors, such as HIF1alpha. ARNTL2 plays important roles in circadian and hypoxia process [10]. Previous studies have explored carcinogenesis roles of ARNTL2 in human malignancies such as breast carcinoma and colorectal adenocarcinoma [11, 12]. In addition, recent researches reported that ARNTL2 is correlated with poor survival and immune infiltration level of lung adenocarcinoma [13, 14]. However, the potential roles of ARNTL2 in ccRCC have not been investigated so far.

In the current study, analysis showed significantly high expression level of ARNTL2 in ccRCC through integrated analyses using RNA-seq data from TCGA and GEO database. The findings were verified in human ccRCC tissue samples and cell lines, highly expressed ARNTL2 was correlated with advanced clinical tumor stage and poor OS. ARNTL2 can also serve as an independent predictor of ccRCC patient survival. Potential biological functions and mechanisms of ARNTL2 were determined through GSEA analysis. A series of in vitro assays were also applied to explore the oncogenic role of ARNTL2 in ccRCC cells. ssGSEA and xCell algorithm were combined and used to analyze the associations between ARNTL2 and TIME in ccRCC. This study sought to explore the roles of ARNTL2 in ccRCC and explore correlation between ARNTL2 expression and tumor immunity.

## Materials and methods

### Data acquisition and analysis

Transcriptome and clinical data of ccRCC (also namely KIRC) patients were retrieved through the Genomic Data Commons (GDC) data portal of TCGA database (https://portal.gdc.cancer.gov/), including 539 ccRCC tissues and 72 normal kidney tissues (in remote kidney parenchyma). The data of GSE15641, GSE46699 and GSE53757 from GEO database (https://www.ncbi.nlm.nih.gov/geo/) were downloaded and used to further validate the expression level of ARNTL2. Genetic mutation data were retrieved from TCGA database and visualization was performed using the package “maftools” in R software [15]. The RNA-seq of ccRCC cell lines were obtained from the Cancer Cell Line Encyclopedia (CCLE) database (https://portals.broadinstitute.org/ccle). IHC staining images of normal kidney tissue sample and kidney cancer tissue sample were downloaded and analyzed from the human protein atlas (HPA) (http://www.proteinatlas.org/) database. All samples were treated with same anti-ARNTL2 antibody (ID: HPA059074).

### Human tissue samples and qRT-PCR analysis

Twenty pairs of ccRCC and adjacent nontumorous tissue samples were gathered from patients who had undergone radical nephrectomy (from 2011 to 2013) at First Affiliated Hospital of Zhejiang University. The adjacent nontumorous tissues were at least 3 cm away from the tumor tissues of ccRCC patients. Informed consent was acquired from every patient and this study was approved by Institutional Ethics Committee in the hospital. All patients’ clinical information was shown in Additional file 1: Table S1. Total RNA was extracted using TRIzol agentia (Takara) and PrimeScript RT Reagent Kit (Takara), RNA was then reverse transcribed into cDNA. The relative ARNTL2 mRNA expression was detected using RT-qPCR, with the help of the ABI 7500 fast real-time PCR System (Applied Biosystems) and SYBR Premix Ex Taq (Takara). GAPDH was used as endogenous normalization reference to quantify the relative mRNA expression of ARNTL2. All primers were listed as follows: ARNTL2 F 5’-ACTTGGTGCTGGTAGTATTGGA-3’; ARNTL2 R 5’-TGTTGGACTCGAATCATC AAGG-3’; GAPDH F 5’-CTGGGCTACACTGAGCACC-3’; GAPDH R 5’- AAGTGGTCGTTGAGGGCAATG-3’.

### Cell lines and cell transfection

Human ccRCC cell lines 786-O, Caki-1, and normal renal cell line HK-2 were obtained from the Shanghai Institute of Cell Biology, Shanghai, China. Cells were cultured in RPMI 1640 medium with 10% fetal bovine serum (FBS) under a humidified atmosphere of 5% CO_2_ at 37℃. The RNA duplexes were chemically synthesized by TsingKe (Hangzhou, China). To enhance the silencing efficiency of siRNA, 3 different small interfering RNAs (siRNA) designed to target ARNTL2 mRNA were merged into a si-ARNTL2 pool to transfect ccRCC cell lines. Polyplus transfection® reagent (Proteintech, Polyplus Transfection) was used for transfection. The 3 siRNA sequences are listed in (Additional file 2: Table S2).

### Western Blot analysis, colony formation and wound healing assay

Western blot analysis was performed as previously reported [16], with the following primary immunoblotting antibodies: anti-GAPDH ((10494-1-AP, Proteintech) and anti-ARNTL2 (ab221557, abcam). After transfection with RNA duplex (50 nM) for 24 h, 786-O or Caki-1 cells were performed colony formation and wound healing assay as previously reported [17].

### Univariate and multivariate cox regression analysis and the construction of nomogram model

Cox regression analyses including univariate and multivariate analysis were employed to identify independent overall survival-related factors. The forest plot was used to show the P value, HR and 95% CI of each variable by using ‘forest plot’ R package. In order to predict the OS possibility of ccRCC individuals, a nomogram was developed based upon the results of multivariate Cox proportional hazards through utilizing the ‘rms’ R package. The Kaplan–Meier plotter analysis (http://kmplot.com/analysis/) were utilized to externally validate the prognostic values of ARNTL2 in ccRCC.

### Gene set enrichment analysis (GSEA)

To investigate the underlying mechanisms and biological functions of ARNTL2, we used the “Cluster Profiler” (version: 3.18.0) package to conduct the GSEA analysis [18] to explore the KEGG signaling pathway analysis of ARNTL2 in ccRCC, The permutation tests were performed 1,000 times, and p-values were adjusted for multiple testing by performing the Benjamini–Hochberg procedure, in the enrichment results, adjust p value < 0.01 and q-values < 0.01 were recognized as a significantly meaningful pathway.

### Tumor immune microenvironment analysis

To perform a reliable assessment of immune infiltration, we preliminarily assess the associations of the expression of ARNTL2 and its top three co-expressed genes with various tumor infiltrated immune cell types by using the ssGSEA algorithm of the GSVA package [19]. xCell algorithm [20] was used to calculate the relative proportions of diverse tumor-infiltrating immune cells in every cancer sample, and the results were implemented using R packages “immunedeconv” and “pheatmap”, we also extracted the expression values of seven most common immune checkpoint-related genes (PD1, PD-L1, CTLA4, TIM3, LAG3, PD-L2 and TIGIT) and assessed the correlations of their expression with ARNTL2 in ccRCC.

### Statistical analysis

Statistical analysis in this study was conducted using R version 4.0.3, SPSS 24.0, and GraphPad Prism 8.0. Wilcox test and Kruskal–Wallis test were utilized to implement the intergroup comparisons of two and three groups, respectively. Spearman correlation analysis was implemented to assess the correlations of ARNTL2 expression with its co-expressed genes in ccRCC tissues and cell lines. For Kaplan–Meier curves, hazard ratio (HR) and p-values were yielded by univariate Cox proportional hazards regression. The predictive model of ARNTL2 was estimated with the receiver operating characteristic curves (ROC) via using pROC package of R software. p < 0.05 denoted statistical significance.

## Results

### ARNTL2 is highly expressed in ccRCC

**Fig. 1:**
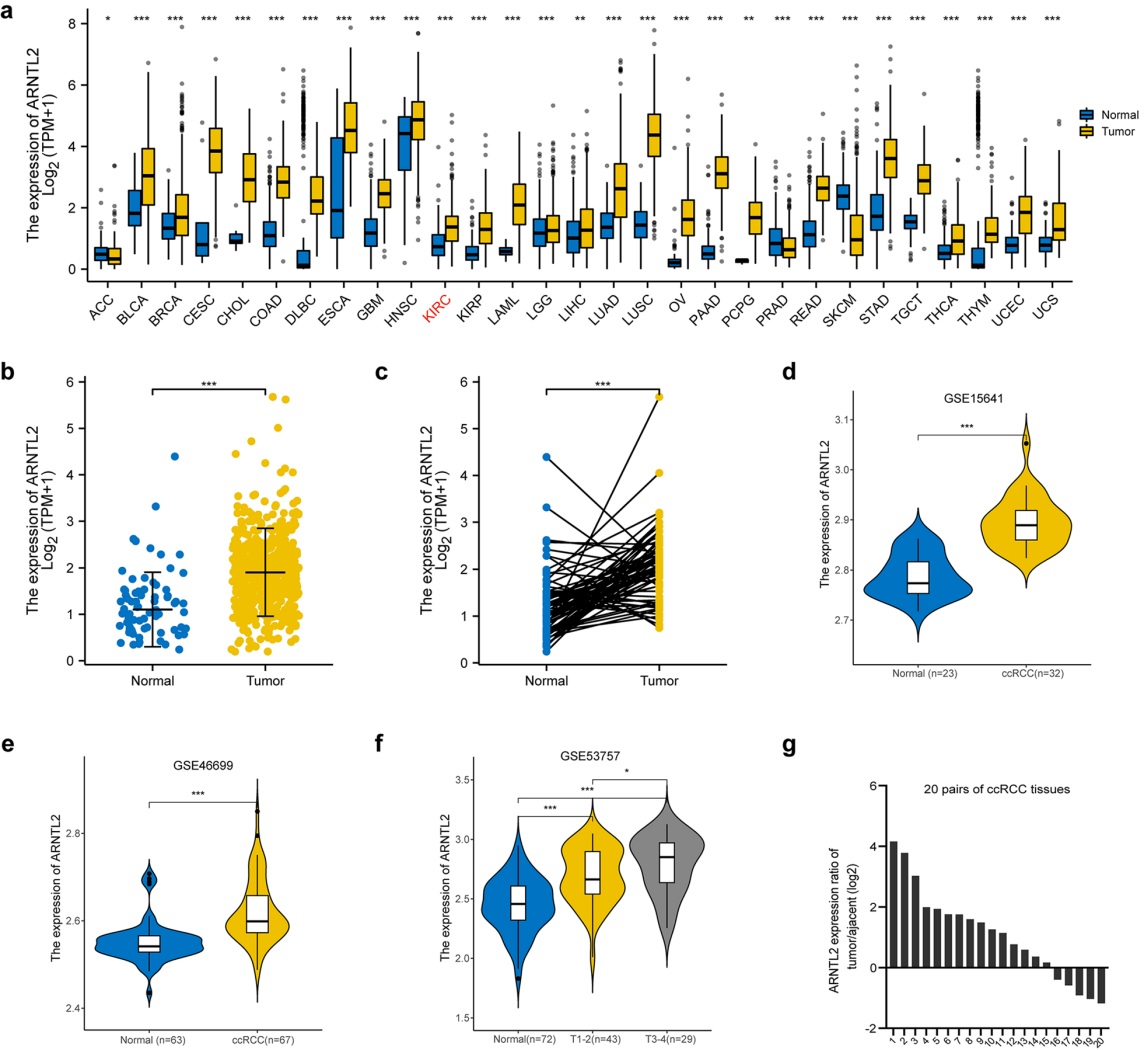
Elevated ARNTL2 expressed in ccRCC. **a** The expression of ARNTL2 in diverse cancer types in TCGA and GTEx dataset. ARNTL2 was overexpressed in ccRCC tissues compared to normal kidney tissues in **b** non-paired (N = 72; T = 539) and **c** paired samples (N = 72; T = 72). The violin plots visualized the relative expression of ARNTL2 in ccRCC in **d** GSE15641, **e** GSE46699 and **f** GSE53757 dataset. **g** The relative expression levels of ARNTL2 in 20 pairs of ccRCC and adjacent normal tissues. *p < 0.05; **p < 0.01, ***p < 0.001

The transcriptional expression level of ARNTL2 in pan-cancer was preliminarily investigated by analyzing the RNA-seq from TCGA and GTEx database. The results indicated that ARNTL2 was significantly upregulated in various cancer types, including ccRCC (Fig. 1a, b). Moreover, a paired line graph of 72 pairs of ccRCC samples and matched nontumorous samples showed that most ccRCC tissues have higher ARNTL2 mRNA expression compared with adjacent normal kidney tissues (Fig. 1c). Three independent datasets from GEO database were utilized to externally illustrate the expression of ARNTL2 in ccRCC, which also demonstrated the highly expressed of ARNTL2 at the transcriptional level in ccRCC (Fig. 1d, e), and increased expression of ARNTL2 significantly associated with the advanced ccRCC clinical stage (Fig. 1f, Table 1) and tumor histologic grade (Table 1), while not significantly associated with age and gender. Additionally, most ccRCC tissues were found to have higher mRNA expression of ARNTL2 compared to adjacent nontumorous tissues in 20 pairs of ccRCC tissues via qRT-PCR method (Fig. 1g). These results revealed that ARNTL2 was significantly elevated in ccRCC.

**Table 1:** Clinicopathological correlation of ARNTL2 expression in human ccRCC

Characteristic	Low expression of ARNTL2	High expression of ARNTL2	p^a^
n	269	270	
Age, n (%)			0.518
<=60	130 (24.1%)	139 (25.8%)	
>60	139 (25.8%)	131 (24.3%)	
Gender, n (%)			0.903
Female	94 (17.4%)	92 (17.1%)	
Male	175 (32.5%)	178 (33%)	
T stage, n (%)			0.027
T1–T2	183 (33.9%)	166 (30.8%)	
T3–T4	86 (16%)	95 (19.3%)	
N stage, n (%)			0.034
N0	119 (46.3%)	122 (47.5%)	
N1	3 (1.2%)	13 (5.1%)	
M stage, n (%)			0.118
M0	220 (43.5%)	208 (41.1%)	
M1	32 (6.3%)	46 (9.1%)	
Pathologic stage, n (%)			0.031
Stage I–II	174 (32.5%)	157 (29.3%)	
Stage III–IV	95 (17.7%)	110 (20.6%)	
Histologic grade, n (%)			0.012
G1–3	233 (43.9%)	223 (42%)	
G4	31 (5.8%)	44 (8.3%)	

^a^p-values were derived from chi-square test

### Multivariate cox regression analysis of ARNTL2 and construction of nomogram model

**Fig. 2:**
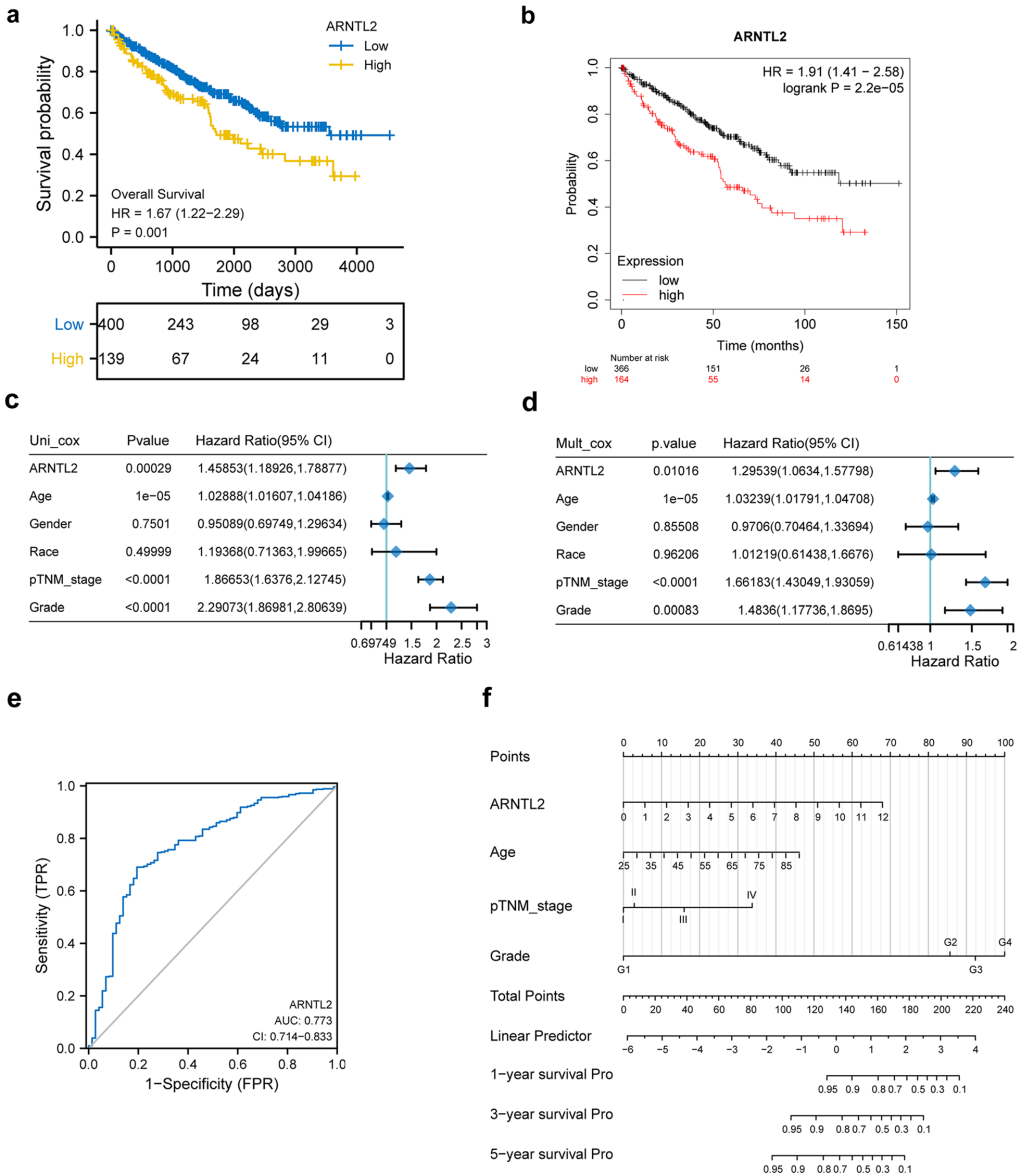
High ARNTL2 expression was an independent predictor of dismal prognosis in ccRCC. **a** The Kaplan–Meier survival curves of the high and low ARNTL2 expression ccRCC patients in TCGA database. **b** The Kaplan–Meier survival curves of the high and low ARNTL2 expression ccRCC patients in Kaplan–Meier Plotter database. **c** Univariate and **d** multivariate Cox regression analysis of ARNTL2 and other clinicopathologic variables with overall survival in ccRCC patients. **e** ROC curve of ARNTL2 in ccRCC. **f** Nomogram was constructed to predict the 1-year, 3-year and 5-year overall survival of ccRCC patients

We then evaluated the prognostic values of ARNTL2 in ccRCC, as plotted in Fig. 2a and b, elevated expression of ARNTL2 predicted poor OS in both TCGA (HR = 1.67, P = 0.001) and Kaplan–Meier plotter (HR = 1.91, P = 2.2e−05) database. The cox analysis was used to explore associations between ARNTL2 expression and OS in ccRCC. Univariate analysis indicated that ARNTL2 expression (HR = 1.45853, p = 0.00029), age (HR = 1.02888, p = 1e−05), pathological TNM stage (HR = 1.86653, p < 0.0001) and tumor grade (HR = 2.29073, p < 0.0001) were significantly correlated with OS of ccRCC (Fig. 2c). Multivariate analysis, depicted as a forest boxplot in Fig. 2d, revealed that ARNTL2 expression (p = 0.01016), the age (p = 1e−05), pathological TNM stage (p < 0.0001), and tumor grade (p = 0.00083) were independent predictor of ccRCC patient OS. ARNTL2 expression also showed preferable predictive ability, as the ROC curve showed that the AUC of ARNTL2 expression for predicting OS was 0.773 (Fig. 2e). Furthermore, we developed a nomogram model by integrating ARNTL2 and other independent prognostic variables base on the multivariate cox regression analysis results (Fig. 2f). The nomogram can contribute to quantitatively assess ccRCC patients 1-y, 3-y and 5-y survival probability.

### Mutation patterns of SMGs in different ARNTL2 expression level of ccRCC

**Fig. 3:**
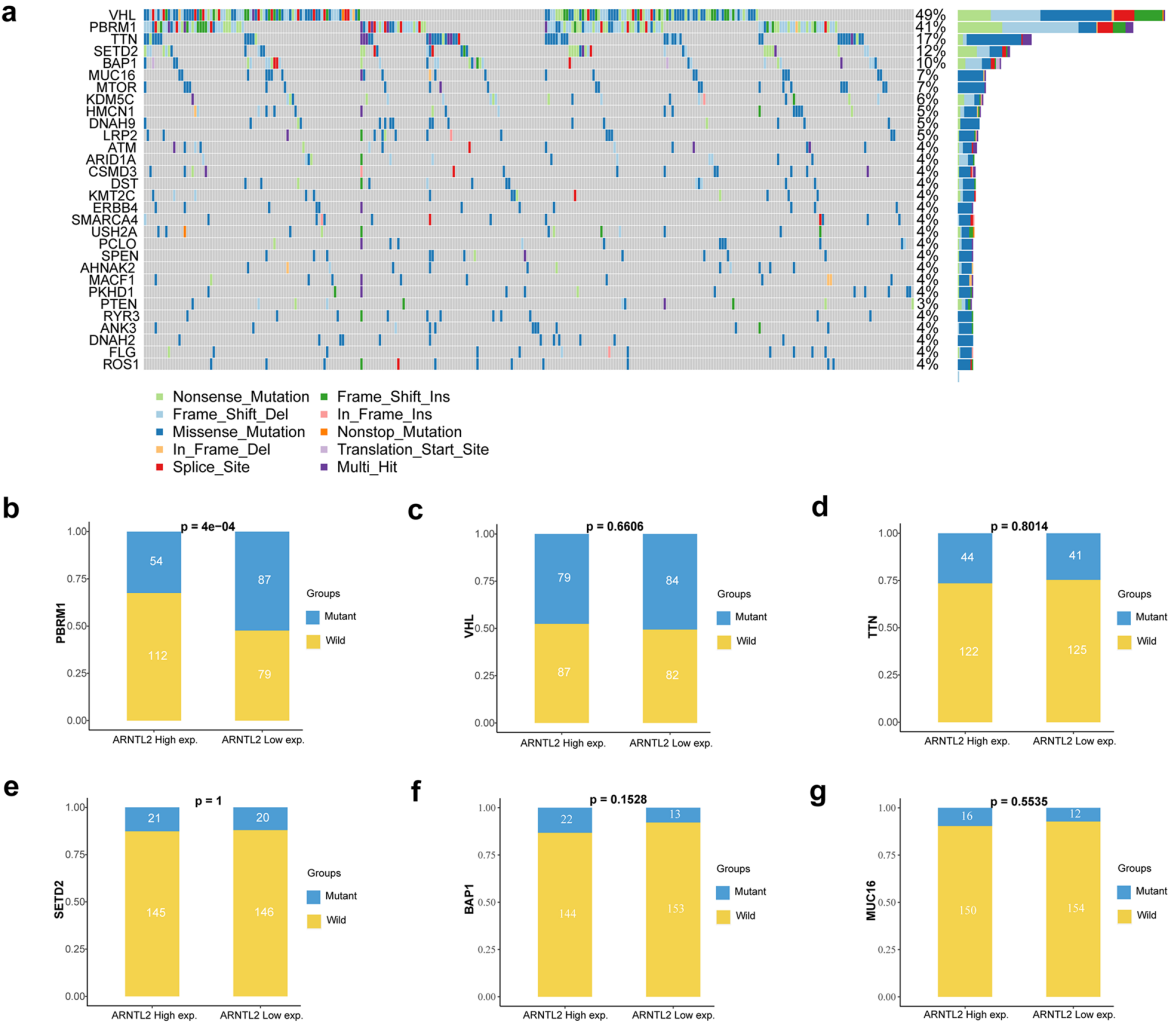
The divergences of significantly mutated genes (SMGs) in ccRCC base on ARNTL2 expression. **a** The mutational landscape of SMGs in ccRCC. The divergence of **b** PBRM1, **c** VHL, **d** TTN, **e** SETD2, **f** BAP1, **g** MUC16 mutation between ARNTL2 high and low ccRCC samples

Researches have reported that genetic mutations are implicated in the development and immune microenvironment of ccRCC [21]. In order to investigate mutation patterns of significantly mutated genes (SMGs) in different ARNTL2 expression level of ccRCC samples, we downloaded and analyzed the genetic mutation data of ccRCC samples from the TCGA database. We identified 30 SMGs in ccRCC cohort, among these, VHL, PBRM1, TTN, SETD2, BAP1, MUC16 were the top six frequently mutated genes in ccRCC (Fig. 3a). Subsequently, we divided the ccRCC patients with genetic mutation data into two groups based upon the median transcriptional expression value of ARNTL2, namely ARNTL2 high and ARNTL2 low group. As shown in Fig. 3b–g, we explore the mutation patterns of top six SMGs in two ARNTL2 groups. Significantly higher mutation frequency of PBRM1 was observed in ARNTL2 low expression group (P < 0.001) (Fig. 3b), while other SMGs were found no differences between two ARNTL2 groups (P > 0.05) (Fig. 3c–g).

### GSEA analysis of ARNTL2 in ccRCC

**Fig. 4:**
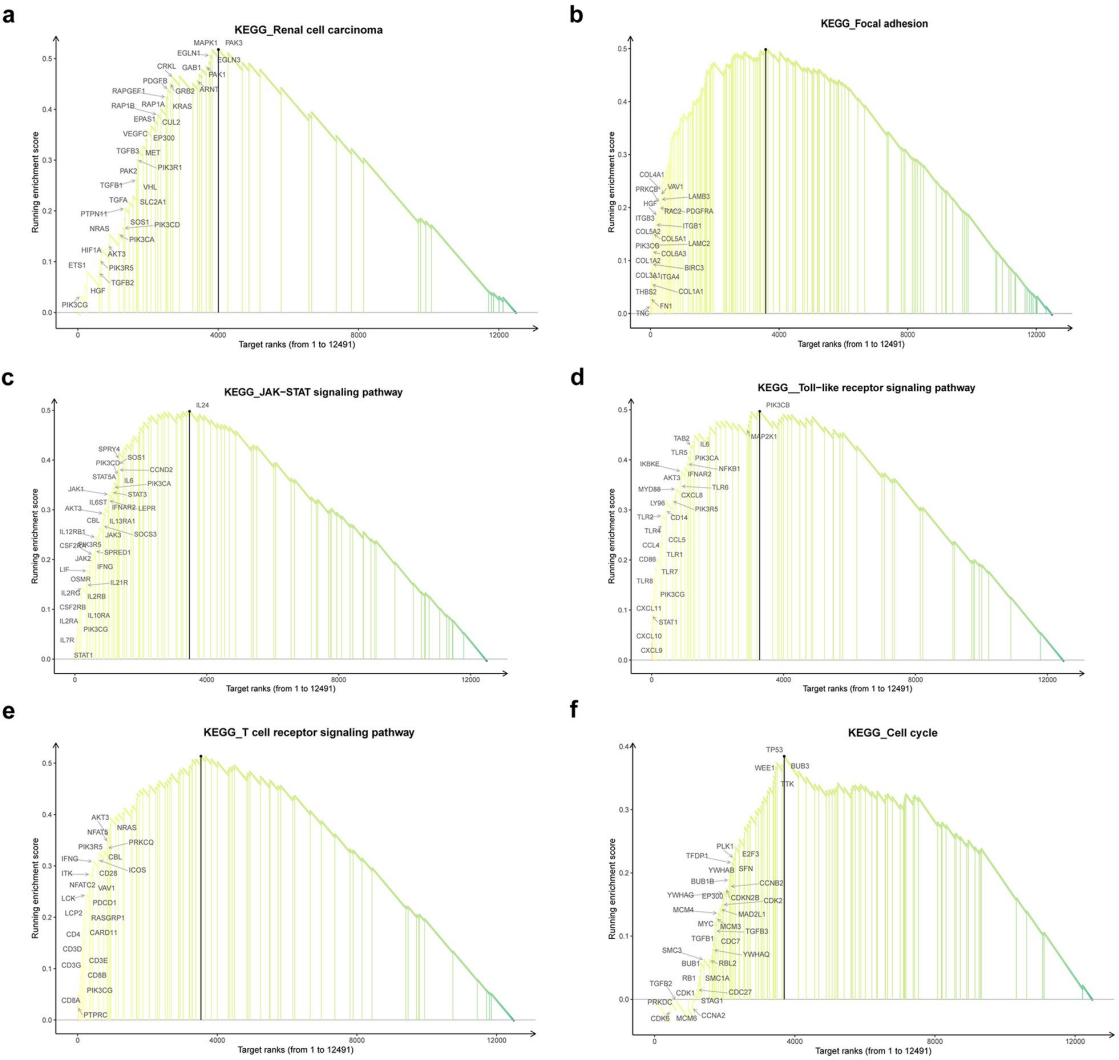
GSEA analyses of ARNTL2 in ccRCC. **a** Renal cell carcinoma. **b** Focal adhesion. **c** JAK-STAT signaling pathway. **d** Toll-like receptor signaling pathway. **e** T cell receptor signaling pathway. **f** Cell cycle

To investigate the potential functions and mechanisms of ARNTL2 in ccRCC, GSEA analysis was applied to explore ARNTL2-related pathways in ccRCC, GSEA analysis contribute to reveal significantly enriched KEGG pathways in highly expressed ARNTL2 samples with a high accuracy. The results revealed that the renal cell carcinoma (NES = 1.96614, P-adjust < 0.001), focal adhesion (NES = 2.06698, P-adjust < 0.001), Toll-like receptor signaling pathway (NES = 1.965199, P-adjust < 0.001), JAK-STAT signaling pathway (NES = 1.999494, P-adjust < 0.001), T cell receptor signaling pathway (NES = 2.036177, P-adjust < 0.001) and cell cycle pathways (NES = 1.551655, P-adjust < 0.005) (Fig. 4a–f, Additional file 3: Table S3) were significantly enriched in upregulated ARNTL2 samples. These results indicated that the immune response and cell cycle related pathways were strongly correlated with abnormal expression of ARNTL2 in ccRCC.

### Depletion of ARNTL2 inhibits ccRCC cells progression in vitro

**Fig. 5:**
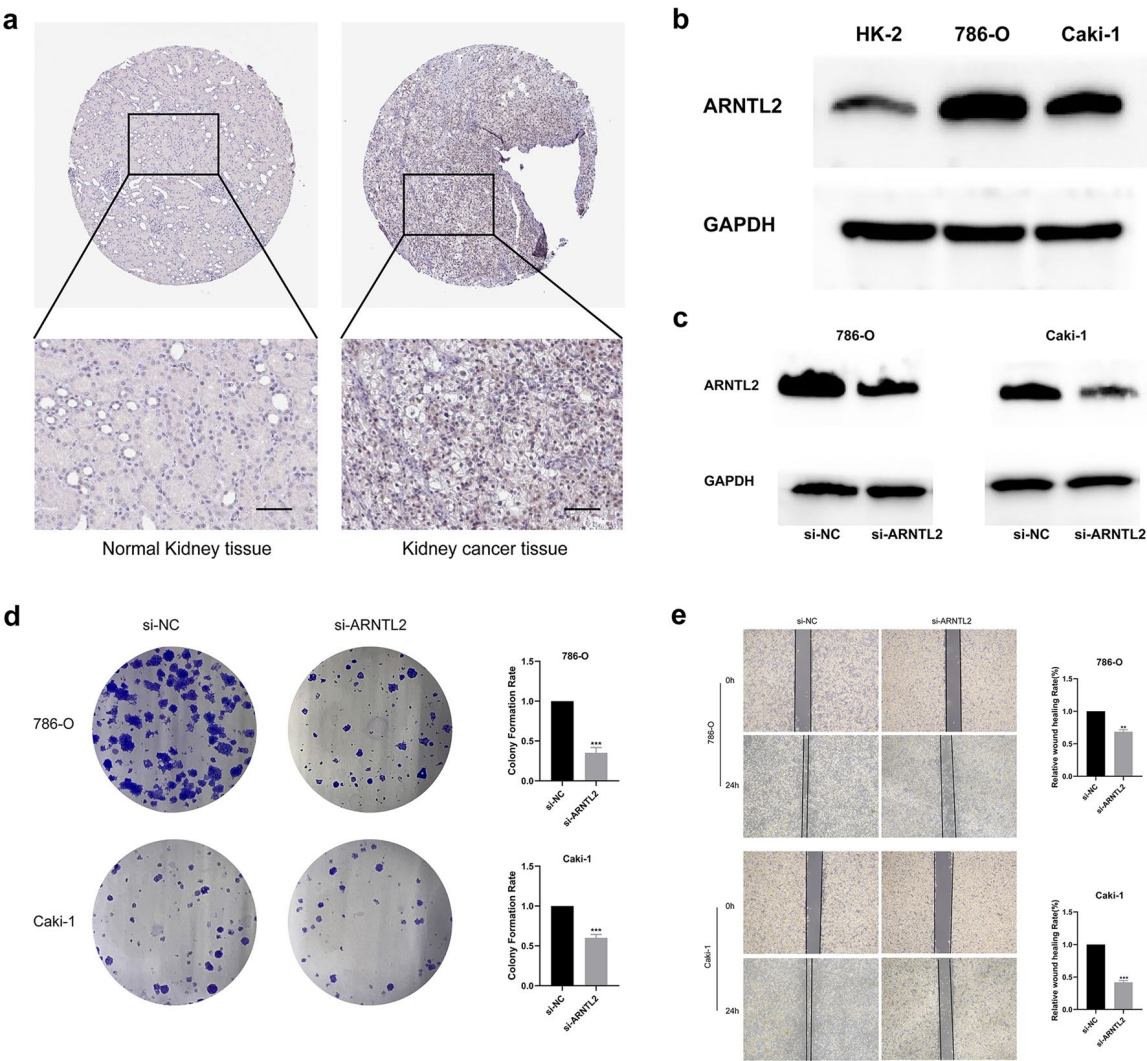
ARNTL2 was upregulated in ccRCC and knockdown of ARNTL2 inhibits ccRCC cells tumorigenicity.** a** The IHC staining of ARNTL2 in normal kidney tissue and kidney cancer tissue were shown (HPA Antibody ID: HPA059074; scale bars, 100 μm). **b** Western blot assay showing the elevated expression of ARNTL2 in ccRCC cell lines (786-O and Caki-1) compared to normal kidney cell line (HK-2). **c** Western blot assay showing the decreased expression of ARNTL2 in ccRCC cell lines after treatment with si-ARNTL2. **d** Colony formation assay demonstrates lacked ARTNL2 suppressed colony formation ability of ccRCC cell lines. **e** The Wound healing assay indicates depletion of ARNTL2 impaired the wound healing efficacy of ccRCC cell lines. **, p < 0.01; ***, p < 0.001

The protein expression level of ARNTL2 were also verified in ccRCC tissue and cell lines (Fig. 5a). Immunohistochemistry analysis showed that ARNTL2 protein expression level is higher in ccRCC tissue compared with the protein expression level in normal kidney tissue. Notably, protein expression level of ARNTL2 was increased in ccRCC 786-O and Caki-1 cell lines compared with HK-2 normal renal cell line (Fig. 5b). To explore the oncogenic role of overexpression ARNTL2 in ccRCC, 786-O and Caki-1 cells were transfected with si-ARNTL2 or si-NC and performed colony formation assay and wound healing assay. The findings showed that knockdown of ARNTL2 (Fig. 5c) significantly suppressed colony formation ability and wound healing efficacy of ccRCC cell lines (Fig. 5d, e).

### Expression of ARNTL2 and its co-expressed genes are significantly correlated with tumor infiltrating immune cells in ccRCC

**Fig. 6:**
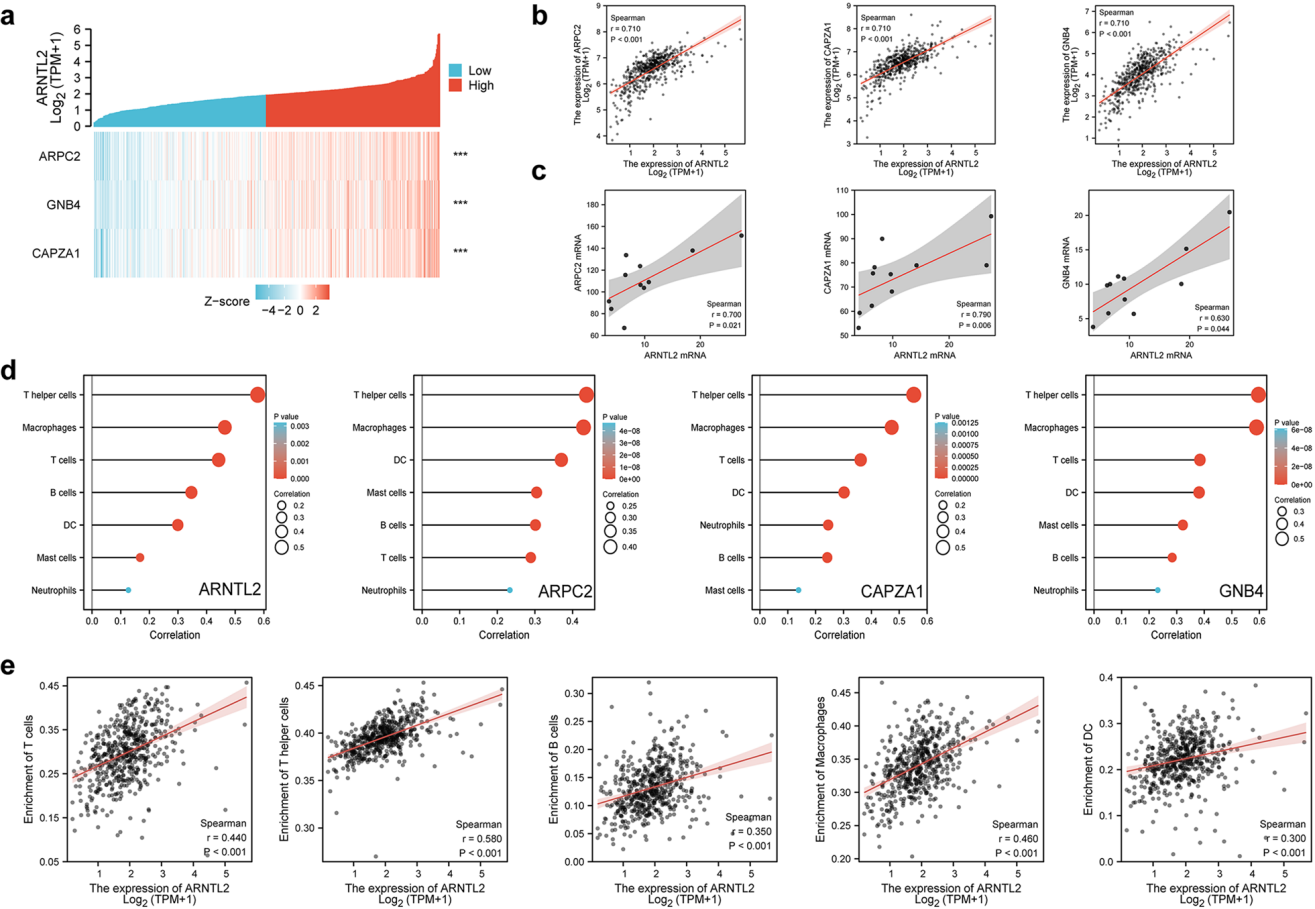
Significant correlations between ARNTL2 and its co-expressed genes and the immune filtration levels in ccRCC. **a** The heatmap visualized the expression patterns of three most co-expressed genes of ARNTL2 in ccRCC. ARNTL2 was remarkably linked to the expression of ARPC2, CAPZA1 and GNB4 in **b** ccRCC tissues and **c** cell lines. **d** Associations of ARNTL2, ARPC2, CAPZA1 and GNB4 expression with cancer-infiltrating immune cell types in ccRCC via ssGSEA analysis. **e** ARNTL2 was significantly linked to the expression level of T cells, T helper cells, B cells, Macrophages and DC in ccRCC

The potential functions of ARNTL2 in the ccRCC carcinogenesis was further explored, we employed R software “stat” package to identify ARNTL2 positively co-expressed genes, and the strongly co-expressed genes (spearman analysis, r > 0.70, p < 0.001) were selected to further analysis, as shown in Fig. 6a, b, the actin related protein 2/3 complex subunit 2 (ARPC2), guanine nucleotide binding protein beta polypeptide 4 (GNB4) and capping-protein muscle Z line alpha 1 (CAPZA1) were the only three positively co-expressed genes with spearman coefficient higher than 0.7 (p < 0.001). We also verified the associations of ARNTL2 with these three genes by analyzing the RNA-seq of ccRCC cell lines in Cancer Cell Line Encyclopedia (CCLE) database, the results demonstrated that these three genes are significantly correlated with the expression of ARNTL2 in ccRCC cell lines (p < 0.05; Fig. 6c). Subsequently, we applied ssGSEA algorithm in R package GSVA to preliminarily estimate the relationships between the expression of ARNTL2 and its top 3 co-expressed genes and tumor infiltrating immune cell types, as shown in Fig. 6d, ARNTL2 and its co-expressed genes ARPC2, GNB4, CAPZA1 were remarkably associated with the infiltrating level of T helper cells, macrophages, T cells, B cells and dendritic cells (DC) (P < 0.001), while showed weak associations with neutrophils and mast cells (P < 0.01). Further correlation analysis revealed that ARNTL2 expression was significantly associated with the enrichment of T cells (r = 0.440, P < 0.001), T helper cells (r = 0.580, P < 0.001), Macrophages (r = 0.460, P < 0.001), B cells (r = 0.350, P < 0.001) and DC (r = 0.300, P < 0.001) (Fig. 6e). In summary, these results indicated that ARNTL2 may participate in the immune response in ccRCC TIME.

**Fig. 7:**
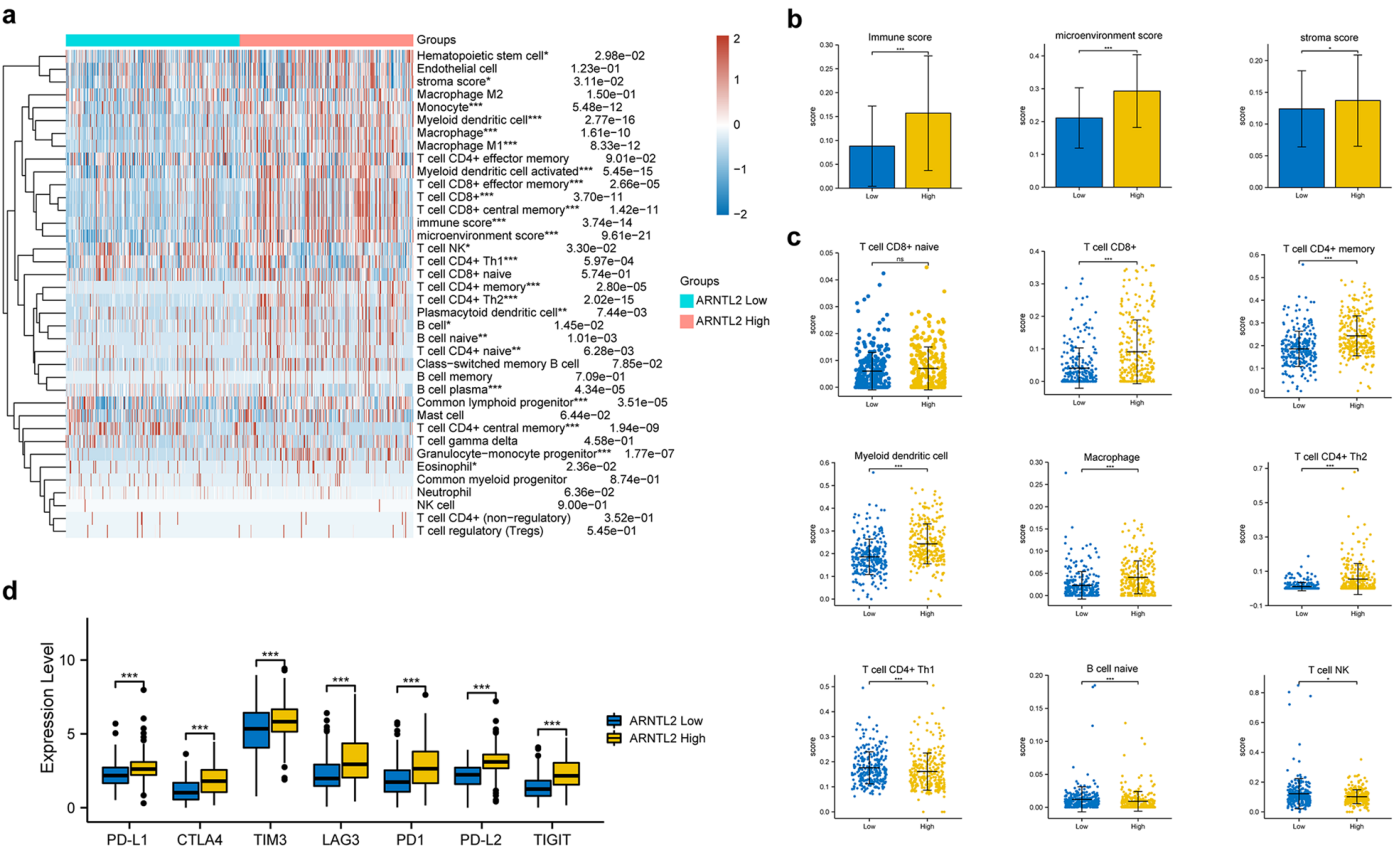
Expression patterns of immune cells and evasion markers in high and low ARNTL2 groups. **a** The heatmap showed significant differences of tumor immune microenvironment between two groups based upon ARNTL2 expression in ccRCC. **b** High ARNTL2 expression correlated with higher immune score, microenvironment score and stroma score compared to low ARNTL2 group. **c** The relative expression level of CD8 + naive T cell, CD8 + cell, CD4 + memory cell, myeloid dendritic cell, macrophage, Th2 CD4 + T cell, Th1 CD4 + T cell, naive B cell, NK T cell. **d** The relative expression level of immune evasion-markers in low and high ARNTL2 expression group

We then divided the ccRCC samples into two groups based on the median mRNA expression value of ARNTL2 in TCGA database, namely ARNTL2 high and ARNTL2 low group. The xCell algorithm was used to calculate the fraction of diverse tumor-infiltrating immune cells in the TIME of high and low ARNTL2 expression ccRCC samples. Distinct infiltrating levels of immune cells were observed in two ARNTL2 groups (Fig. 7a). With the rise of the ARNTL2 expression, the immune score (P < 0.001), microenvironment score (P < 0.001) and stroma score (P < 0.05) in ccRCC TIME were enhanced (Fig. 7b), high expression level of ARNTL2 tightly linked to the infiltrating levels of CD8 + T cell, CD4 + memory T cell, Myeloid dendritic cell, macrophage and CD4 + Th2 T cell. However, CD4 + Th1 T cell, B naive cell and NK T cell were downregulated in highly ARNTL2 expression group (Fig. 7c). Additionally, the immune checkpoint-related genes, such as PD-L1, PD-L2, CTLA4, PD-1, TIM3, LAG3 and TIGIT were also tightly related to the expression level of ARNTL2 (Fig. 7d). These findings showed that ARNTL2 is implicated in T cell exhaustion and immune evasion in ccRCC.

## Discussion

Although significant advances have been reported in therapeutic strategies such as surgical excision, chemotherapy and targeted therapies of ccRCC in recent years, ccRCC severely affects the health and quality of life of patients worldwide [3]. Immunotherapy is a recent approach developed for advanced ccRCC patients, especially for patients who cannot tolerate chemotherapy. However, most patients are unable to obtain durable benefits from current immunotherapy represented by anti-PD-1 antibodies therapy [22, 23]. It is urgent to identify effective biomarkers and immune-related therapeutic targets to improve the survival of patients with ccRCC. The findings of the current study showed that ARNTL2 was significantly upregulated in ccRCC tissues and cell lines compared with normal kidney tissues and cell line, overexpression of ARNTL2 could predict unfavorable prognosis for ccRCC patients, a series of analysis and in vitro assays were further conducted to explore the oncogenic role of ARNTL2 and its associations with immune infiltration in ccRCC [10].

The potential roles of ARNTL2 has been explored in several human malignancies. Mazzoccoli et al. discovered that ARNTL2 was upregulated in colorectal cancer specimens and could serve as an independent predictor of colorectal cancer patient survival [12]. Brady et al. demonstrated that highly expressed transcription factor ARNTL2 was associated with dismal survival of lung adenocarcinoma patients [24]. However, the clinical values and potential functions of ARNTL2 in ccRCC remains elusive. In this study, we carried out a systematical analysis by integrating available data from GEO and TCGA database, and validated the overexpression of ARNTL2 in human ccRCC clinical tissues and cell lines. Overexpressed ARNTL2 correlated with advanced clinical tumor stages and grades, we also found that upregulation of ARNTL2 predicted poor overall survival and can serve as an independent prognostic variable in ccRCC. Additionally, in order to contribute to clinical decision making, a nomogram was subsequently developed based upon the results of multivariate Cox analysis including the expression level of ARNTL2 in ccRCC. The oncogenic role of ARNTL2 in human cancer was firstly illustrated in hepatocellular carcinoma, Yeh et al. demonstrated that overexpression of ARNTL2 promotes cancer cell proliferation [25]. Subsequently, Brady et al. showed that ARNTL2 can drive lung adenocarcinoma metastasis [24]. Additionally, Ha et al. demonstrated that ARNTL2 is essential for breast cancer metastasis [11]. In the present study, we performed a series of in vitro assays, and found that depletion of ARNTL2 significantly suppressed the colony formation ability and wound healing efficacy of ccRCC cell lines, our results indicated that ARNTL2 might plays a pivotal role in ccRCC progression.

Recent studies report that PBRM1 is the second-most commonly mutated gene behind VHL in ccRCC [26, 27]. In the present study, genetic mutation information of ccRCC patients were retrieved from TCGA database and analyzed. The findings showed that PBRM1 was ranked second among 30 significantly mutated genes (SMGs) in ccRCC. Notably, the findings showed that PBRM1 mutation frequency was significantly higher in ARNTL2 low group compared with ARNTL2 high group, whereas other SMGs showed no significant differences in mutation frequency between the two groups. Miao et al. reported that ccRCC patients with high PBRM1 mutation frequency could benefit more from immune checkpoint inhibitor (ICI) therapy compared with those with low mutation frequency [5], Braun et al. preformed a randomized clinical trial and reported that PBRM1 mutations can be used as a marker of ICI response in ccRCC [28]. These findings imply that low ARNTL2 expression ccRCC patients with high PBRM1 mutation rates can achieve more clinical benefits from ICI therapy. Furthermore, recent studies reported that ARNTL2 is correlated with prognosis and immune infiltration of lung adenocarcinoma [13, 14]. These studies indicate that ARNTL2 may affect the TIME of cancer. The GSEA results indicated that ARNTL2 is implicated in several signaling pathways, including T cell receptor signaling pathway, renal cell carcinoma, focal adhesion, JAK-STAT signaling pathway, and cell cycle pathway. JAK-STAT signaling pathway is implicated in carcinogenesis and immune infiltration of ccRCC [29, 30]. Fang et al. reported that simvastatin inhibits the growth and metastasis of renal cancer cell through targeting JAK2/STAT3, ERK and AKT/mTOR pathway [31]. Miao et al. reported changes in JAK-STAT and immune signaling pathways in PBRM1-deficent renal cancer cells [5]. These findings revealed that ARNTL2 may affect the TIME of ccRCC by activating JAK-STAT signaling pathway.

To explore the potential roles of ARNTL2 in the TIME of ccRCC, we initially assessed the associations of the expression of ARNTL2 and its top three co-expressed genes with various tumor infiltrated immune cell types in ccRCC. The results revealed that ARNTL2 and its co-expressed genes were remarkably linked to the enrichment of T cells, T helper cells and macrophages in ccRCC. Furthermore, xCell algorithm analysis showed significant differences in immune score, microenvironment score, stroma score, infiltrating immune cell types and immune-evasion marker genes such as PD-1, PD-L1 expression between high and low ARNTL2 group. High ARNTL2 expression was correlated with high infiltration level of CD8 + T cell, macrophage, DC and Th2 CD4 + T cell, and overexpression of immune escape-related genes such as PD-1, PD-L1, PD-L2, CTLA4. On the other hand, the low ARNTL2 expression group exhibited high infiltration level of CD4 + Th1 T cell and NK T cells. Clark et al. utilized the xCell algorithm and combined molecular characteristics to stratify ccRCC into four immune subtypes. Among the four subtypes, CD8 + inflamed subtype was correlated with the worst prognosis and was characterized by a highly infiltrated level of CD8 + T cell, and overexpressed immune-escape genes such as PD1, PD-L1, PD-L2, and CTLA4 [32]. Braun et al. reported that high infiltration level of CD8 + T cell was associated with poor prognosis of patients with ccRCC [33]. These findings indicated that ARNTL2 may be involved in the formation of CD8 + inflamed ccRCC subtype and immune escape.

Several limitations also merit attentions. Firstly, associations between TIME and ARNTL2 were analyzed only based on TCGA database due to paucity of applicable data in our own cohort, although we illustrated the correlations through multiple methods. Further reliable clinical trials with larger samples should be conducted to validate these findings. Additionally, the underlying regulatory mechanisms of ARNTL2 in ccRCC should be elucidated through basic studies and clinical trials.

## Conclusions

The findings of this study showed that the high expression level of ARNTL2 is correlated with unfavorable prognosis and immune infiltration in ccRCC. Furthermore, ARNTL2 can be used as an independent prognostic marker for ccRCC, and depletion of ARNTL2 inhibited ccRCC cells progression in vitro. Moreover, the interactions between ARNTL2 and focal adhesion pathway, JAK-STAT signaling pathway, cell cycle pathway, Toll-like receptor pathway and T cell receptor signaling pathway may be implicated in carcinogenesis and regulation of TIME of ccRCC. High expression of ARNTL2 exhibited immune infiltration status similar to CD8 + inflamed ccRCC subtype, which was characterized by high infiltration level of CD8 + T cell and high expression of immune-escape genes such as PD1, PD-L1, PD-L2, and CTLA4. Further deep studies based on in vivo and in vitro basic experiments and randomized clinical trials using large samples should be conducted to validate the findings of this study.

## Supplementary Information

**Additional file 1: Table S1.** The patients’ clinical information(n=20) in this study.

**Additional file 2: Table S2.** Oligonucleotide sequences of si-ARNTL2.

**Additional file 3: Table S3.** Gene set enrichment analysis (GSEA) of ARNTL2 in ccRCC.

## Data Availability

The datasets exhibited in present study can be discovered in online repositories, further inquiries can be directed to the corresponding author.

## Abbreviations

GEO: Gene expression omnibus
ARNTL2: Aryl hydrocarbon receptor nuclear translocator-like 2
TCGA: The cancer genome atlas
GSEA: Gene set enrichment analysis
GTEx: Genotype-tissue expression
TIME: Tumor immune microenvironment
TMB: Tumor mutational burden
OS: Overall survival
MSI: Microsatellite instability
ccRCC: Clear cell renal cell carcinoma
PD-L1: Programmed cell death ligand 1
CTLA4: Cytotoxic T-lymphocyte antigen 4
LAG3: Lymphocyte-activation gene 3
PD1: Programmed cell death 1
TIM3: T cell immunoglobulin domain and mucin domain 3
PD-L2: Programmed death ligand 2
TIGIT: T cell immunoglobulin and ITIM domain
ROC: Receiver operating characteristic curve
SMGs: Significantly mutated genes
ARPC2: Actin related protein 2/3 complex subunit 2
GNB4: Guanine nucleotide binding protein beta polypeptide 4
CAPZA1: Capping protein muscle Z line alpha 1
UCS: Uterine carcinosarcoma
SARC: Sarcoma
COAD: Colon adenocarcinoma
PAAD: Pancreatic adenocarcinoma
BRCA: Breast invasive carcinoma
LGG: Brain lower grade glioma
BLCA: Bladder urothelial carcinoma
OV: Ovarian serous cystadenocarcinoma
STAD: Stomach adenocarcinoma
LUAD: Lung adenocarcinoma
UVM: Uveal melanoma
ESCA: Esophageal carcinoma
MSH2: MutS homolog 2
LUSC: Lung squamous cell carcinoma
MESO: Mesothelioma
ACC: Adrenocortical carcinoma
MLH1: MutL homolog 1
TGCT: Testicular germ cell tumors
READ: Rectum adenocarcinoma
LAML: Acute myeloid leukemia
STAD: Stomach adenocarcinoma
MSH6: MutS homolog 6
DLBC: Lymphoid neoplasm diffuse large B-cell lymphoma
CHOL: Cholangiocarcinoma
HNSC: Head and neck squamous cell carcinoma
PCPG: Pheochromocytoma and paraganglioma
GBMLGG: Glioblastoma multiforme and lower grade glioma
PMS2: Post meiotic segregation increased 2
