# Supplementary material for: Upregulation of ARNTL2 is associated with poor survival and immune infiltration in clear cell renal cell carcinoma
Source: Cancer Cell Int. 2021 Jul 3;21:341. doi: 10.1186/s12935-021-02046-z (PMC8255002; doi:10.1186/s12935-021-02046-z)
Supplement: Supplementary file 1 — Additional file 1: Table S1. The patients’ clinical information(n=20) in this study. [file 12935_2021_2046_MOESM1_ESM.docx]

| **Table S1. The patients’ clinical information(n=20) in this study.** | | | | | | |
| --- | --- | --- | --- | --- | --- | --- |
| No. | Age | Sex Pathological diagnosis | | pT Stage | Fuhrman Grade | |
| 1 | 65 | male | ccRCC | T1b | I |  |
| 2 | 51 | female | ccRCC | T1b | II |  |
| 3 | 67 | male | ccRCC | T2a | I |  |
| 4 | 53 | female | ccRCC | T1b | I |  |
| 5 | 56 | male | ccRCC | T2a | III |  |
| 6 | 57 | male | ccRCC | T1a | I |  |
| 7 | 54 | male | ccRCC | T3a | II |  |
| 8 | 65 | female | ccRCC | T1b | I |  |
| 9 | 63 | female | ccRCC | T1b | II |  |
| 10 | 60 | male | ccRCC | T4 | II |  |
| 11 | 54 | female | ccRCC | T3a | I |  |
| 12 | 58 | female | ccRCC | T1b | I |  |
| 13 | 59 | male | ccRCC | T3b | III |  |
| 14 | 43 | male | ccRCC | T1a | I |  |
| 15 | 75 | male | ccRCC | T1a | I |  |
| 16 | 62 | male | ccRCC | T1b | II |  |
| 17 | 74 | male | ccRCC | T1b | I |  |
| 18 | 68 | female | ccRCC | T3a | II |  |
| 19 | 59 | female | ccRCC | T1b | I |  |
| 20 | 55 | female | ccRCC | T1b | II |  |
