# Supplementary material for: Upregulation of ARNTL2 is associated with poor survival and immune infiltration in clear cell renal cell carcinoma
Source: Cancer Cell Int. 2021 Jul 3;21:341. doi: 10.1186/s12935-021-02046-z (PMC8255002; doi:10.1186/s12935-021-02046-z)
Supplement: Supplementary file 2 — Additional file 2: Table S2. Oligonucleotide sequences of si-ARNTL2. [file 12935_2021_2046_MOESM2_ESM.docx]

| **Table S2. Oligonucleotide sequences of si-ARNTL2.** | | | |
| --- | --- | --- | --- |
| si-RNA | sense（5'-3'） |  | antisense（5'-3'） |
| si-ARNTL2-1 | GAUUACAGCCAUAUAUUGUTT | | ACAAUAUAUGGCUGUAAUCTT |
|  |  |  |  |
| si-ARNTL2-2 | GGUAGUAUUGGAACAGAUATT | | UAUCUGUUCCAAUACUACCTT |
|  |  |  |  |
| si-ARNTL2-3 | CUUAUGUGGGAAGUAAUUATT | | UAAUUACUUCCCACAUAAGTT |
