# Supplementary material for: Upregulation of ARNTL2 is associated with poor survival and immune infiltration in clear cell renal cell carcinoma
Source: Cancer Cell Int. 2021 Jul 3;21:341. doi: 10.1186/s12935-021-02046-z (PMC8255002; doi:10.1186/s12935-021-02046-z)
Supplement: Supplementary file 3 — Additional file 3: Table S3. Gene set enrichment analysis (GSEA) of ARNTL2 in ccRCC. [file 12935_2021_2046_MOESM3_ESM.docx]

| **Table S3. Gene set enrichment analysis (GSEA) of ARNTL2 in ccRCC.** | | | |
| --- | --- | --- | --- |
| GeneSet name | NES^*^ | p-adjust | q-values |
| KEGG_FOCAL_ADHESION | 2.06698 | 0.000503 | 0.000288 |
| KEGG_JAK_STAT_SIGNALING_PATHWAY | 1.999494 | 0.000503 | 0.000288 |
| KEGG_T_CELL_RECEPTOR_SIGNALING_PATHWAY | 2.036177 | 0.000503 | 0.000288 |
| KEGG_TOLL_LIKE_RECEPTOR_SIGNALING_PATHWAY | 1.965199 | 0.000503 | 0.000288 |
| KEGG_RENAL_CELL_CARCINOMA | 1.96614 | 0.000503 | 0.000288 |
| KEGG_CELL_CYCLE | 1.551655 | 0.002661 | 0.001522 |
| *NES: Normalized Enrichment Score. | | | |
